# No Correlation between Biofilm Formation, Virulence Factors, and Antibiotic Resistance in *Pseudomonas aeruginosa*: Results from a Laboratory-Based In Vitro Study

**DOI:** 10.3390/antibiotics10091134

**Published:** 2021-09-20

**Authors:** Márió Gajdács, Zoltán Baráth, Krisztina Kárpáti, Dóra Szabó, Donatella Usai, Stefania Zanetti, Matthew Gavino Donadu

**Affiliations:** 1 Department of Oral Biology and Experimental Dental Research, Faculty of Dentistry, University of Szeged, Tisza Lajos körút 63, 6720 Szeged, Hungary; 2 Institute of Medical Microbiology, Faculty of Medicine, Semmelweis University, Nagyvárad tér 4, 1089 Budapest, Hungary; szabo.dora@med.semmelweis-univ.hu; 3 Department of Prosthodontics, Faculty of Dentistry, University of Szeged, Tisza Lajos körút 62–64, 6720 Szeged, Hungary; barzol34@gmail.com; 4 Department of Orthodontics and Pediatric Dentistry, Faculty of Dentistry, University of Szeged, Tisza Lajos körút 62–64, 6720 Szeged, Hungary; drkarpatik@gmail.com; 5 Department of Biomedical Sciences, University of Sassari, 07100 Sassari, Italy; dusai@uniss.it (D.U.); zanettis@uniss.it (S.Z.); mdonadu@uniss.it (M.G.D.); 6 Department of Chemistry and Pharmacy, University of Sassari, 07100 Sassari, Italy

**Keywords:** *Pseudomonas aeruginosa*, antimicrobial resistance, biofilm, pigment, motility

## Abstract

*Pseudomonas aeruginosa* (*P. aeruginosa*) possesses a plethora of virulence determinants, including the production of biofilm, pigments, exotoxins, proteases, flagella, and secretion systems. The aim of our present study was to establish the relationship between biofilm-forming capacity, the expression of some important virulence factors, and the multidrug-resistant (MDR) phenotype in *P. aeruginosa.* A total of three hundred and two (n = 302) isolates were included in this study. Antimicrobial susceptibility testing and phenotypic detection of resistance determinants were carried out; based on these results, isolates were grouped into distinct resistotypes and multiple antibiotic resistance (MAR) indices were calculated. The capacity of isolates to produce biofilm was assessed using a crystal violet microtiter-plate based method. Motility (swimming, swarming, and twitching) and pigment-production (pyoverdine and pyocyanin) were also measured. Pearson correlation coefficients (r) were calculated to determine for antimicrobial resistance, biofilm-formation, and expression of other virulence factors. Resistance rates were the highest for ceftazidime (56.95%; n = 172), levofloxacin (54.97%; n = 166), and ciprofloxacin (54.64%; n = 159), while lowest for colistin (1.66%; n = 5); 44.04% (n = 133) of isolates were classified as MDR. 19.87% (n = 60), 20.86% (n = 63) and 59.27% (n = 179) were classified as weak, moderate, and strong biofilm producers, respectively. With the exception of pyocyanin production (0.371 ± 0.193 vs. non-MDR: 0.319 ± 0.191; *p* = 0.018), MDR and non-MDR isolates did not show significant differences in expression of virulence factors. Additionally, no relevant correlations were seen between the rate of biofilm formation, pigment production, or motility. Data on interplay between the presence and mechanisms of drug resistance with those of biofilm formation and virulence is crucial to address chronic bacterial infections and to provide strategies for their management.

## 1. Introduction

*Pseudomonas aeruginosa* (*P. aeruginosa*) is a motile, non-fermenting Gram-negative bacillus with non-fastidious growth requirements, which is ubiquitous in various aquatic environments, in addition to being commonly involved in healthcare-associated infections (HAIs) [1,2]. *P. aeruginosa* is considered as an opportunistic pathogen (8–20% of hospitalized individuals are colonized), as it is more commonly found in patients affected by invasive surgical interventions, immunosuppression (associated with malignancies and their treatment, HIV infection), or other underlying diseases (e.g., diabetes) [3,4,5]. This microorganism has been associated with a wide variety of hard-to-treat infections, such as ventilator-associated pneumonia (VAP), sepsis, skin and soft tissue infections (linked to burn injuries or pressure ulcers), bone and joint infections, otitis externa, and keratitis [6,7]; in addition, multisite infections are also fairly common. *Pseudomonas* spp. are also frequent colonizers of the airways in patients affected by cystic fibrosis, contributing to acute exacerbations and the progressive decrease in lung function [8].

Although *P. aeruginosa* (and non-fermenters in general) is considered a low-grade pathogen, it possesses a plethora of virulence determinants, including its production of pigments (pyoverdine, pyocyanine and pyomelanin), exotoxins (such as exotoxins A, S, T and U), proteases (leading to tissue destruction), siderophores, lectins, flagella, and various secretion systems (most notably the Type III “injectasome”) [9,10]. However, the production of biofilm is probably the most important factor in the survival of *P. aeruginosa* in harsh environmental conditions (such as in nosocomial settings, like intensive care units [ICUs] and surgical theaters), facilitating the establishment of chronic infections and persistence in vivo [11,12]. Biofilms are composed on mono- or multispecies aggregates of bacterial communities, various exopolysaccharides (EPS), environmental DNA, and other biomolecules (lipids, proteins, carbohydrates) [13]. Biofilms provide protection from sheer forces, drying and immune cells, while also leading to the adaptation of *P. aeruginosa* into metabolically-inactive persister cells [14]. In addition to persister (or small colony variant; SCV) formation, the chemical composition of biofilms inhibits the diffusion of antimicrobials (acting as a pharmacokinetic barrier to these drugs) and disinfectants/biocides [15]. Consequently, the minimum inhibitory concentrations (MICs) of bacteria embedded in biofilm may be 10^1^–10^4^ times higher, compared to their planktonic counterparts [16].

The emergence of antimicrobial resistance (AMR) has become a critical issue for healthcare professionals worldwide, as their therapeutic armamentarium has become severely limited to address infections caused by multidrug-resistant (MDR) bacteria [17,18]. As a consequence, infections caused by MDR pathogens are associated with increased mortality rates and hospitalization costs, and decreased quality of life (QoL) in affected patients [19]. *P. aeruginosa* possesses intrinsic resistance to a wide range of antimicrobials, and due to its pronounced genomic plasticity and rich resistome, it has a particular propensity of acquire mechanisms of resistance (through horizontal gene transfer) to several, structurally-distinct antimicrobial drugs [9,20]. As a result, *P. aeruginosa* isolates with high-level resistance to fluoroquinolones, aminoglycosides, and carbapenems are increasingly common worldwide [21]. The association between biofilm-forming capacity, virulence factor expression, and the MDR phenotype in pathogenic bacteria has been studied extensively [22,23]; however, the topic is still a contentious issue, as many authors—using various methodologies and involving different species of clinically-relevant bacteria—have come to markedly different conclusions. With this in mind, the aim of our present study was to establish the relationship between biofilm-forming capacity, the expression of some important virulence factors, and the MDR phenotype in *P. aeruginosa*.

## 2. Materials and Methods

### 2.1. Isolates

A total of three hundred and two (n = 302) *P. aeruginosa* isolates were included in this study, which were kindly provided from the strain collections of a Hungarian and Italian tertiary care hospital. The study uses a cross-sectional design, with microorganisms that were isolated between 1 Janurary 2019 and 1 Janurary 2020, originating from various types of clinical specimens, being randomly selected to be included in our experiments. During the study, *P. aeruginosa* ATCC 27853 (MDR isolate, weak biofilm producer) and *P. aeruginosa* PAE 170022 (susceptible isolate, strong biofilm producer) were used as control strains, obtained from the American Type Culture Collection (Manassas, VI, USA) [24]. Stock cultures were stored at −80 °C in a cryopreservation medium (700 µL trypticase soy broth + 300 µL 50% glycerol) until use.

### 2.2. Re-Identification of Isolates

All isolates included in our study were re-identified as P. aeruginosa before further experiments. Re-identification of isolates was carried out using matrix-assisted laser desorption/ionization time-of-flight mass spectrometry (MALDI-TOF MS; Bruker Daltonics, Bremen, Germany); to perform the MALDI-TOF assay, bacterial cells from fresh overnight cultures on agar plates were transferred to a stainless steel target with a sterile toothpick. An on-target extraction was performed by adding 1 µL of 70% formic acid prior to the matrix. After drying at room temperature, the cells were covered with 1 µL matrix (α-cyano-4-hydroxy cinnamic acid in 50% acetonitrile/2.5% trifluoro-acetic acid; Bruker Daltonics, Bremen, Germany). Mass spectrometry analyses were performed by a MicroFlex MALDI Biotyper (Bruker Daltonics, Bremen, Germany) in positive linear mode across the m/z range of 2 to 20 kDa; for each spectrum, 240 laser shots at 60 Hz in groups of 40 shots per sampling area were collected [22]. For analyses of spetra, the MALDI Biotyper RTC 3.1 software and the MALDI Biotyper Library 3.1 (Bruker Daltonics. Bremen. Germany) were utilized. After analysis, a log(score) value was assigned to all isolates, indicating the reliability of MALDI-TOF MS identification. The log(score) values were evaluated as follows: a log(score) <1.69 showed unreliable identification, 1.70–1.99 corresponded to probable genus-level identification, 2.00–2.29 corresponded to reliable genus-level identification, while a score ≥2.30 corresponded to reliable species-level identification [25].

### 2.3. Antimicrobial Susceptibility Testing, Resistotyping

Antimicrobial susceptibility testing for respective isolates was carried out using the Kirby-Bauer disk diffusion method (Oxoid, Basingstoke, UK), and in subsequent experiments (when relevant) with E-tests (Liofilchem, Roseto degli Abruzzi, Italy) on Mueller-Hinton agar (MHA) (bioMérieux, Marcy-l’Étoile, France) plates in case of ceftazidime (CAZ; 10 µg), cefepime (FEP; 30 µg), imipenem (IMI; 10 µg), meropenem (MER; 10 µg), ciprofloxacin (CIP; 5 µg), levofloxacin (LEV; 5 µg), gentamicin (GEN; 10 µg), and amikacin (AMI; 30 µg), respectively. Colistin (COL) susceptibility was performed using the broth microdilution method in cation-adjusted Mueller–Hinton broth (MERLIN Diagnostika GmbH, Bremen, Germany). Intermediate results were grouped with and reported as resistant [26]. The isolates were grouped into distinct resistotypes based on the presence of phenotypic resistance to relevant antimicrobials [27]. Interpretation of the results were based on the recommendations of the European Committee on Antimicrobial Susceptibility Testing (EUCAST) relevant at the time of isolation [28]. Classification of the isolates as (MDR; resistance to at least one agent in ≥3 antibiotic groups) was based on Magiorakos et al. [29]; in subsequent analyses, isolates were divided as non-MDR and MDR. A multiple antibiotic resistance (MAR) index—ranging between 0 and 1—was calculated by dividing the total number of detected resistance to antimicrobials for each isolate by the total number of tested antimicrobials [27].

### 2.4. Phenotypic Detection of AmpC Overexpression and Carbapenemase Production

In CAZ-resistant isolates, the overexpression of AmpC β-lactamase enzymes was tested by an agar plate method, where the agar base was supplemented with cloxacillin (250 µg/mL); during this assay, the property of cloxacillin—capable of inhibiting the effects of AmpC β-lactamases—was used [26,30]. A two-fold difference in CAZ MICs (determined by E-tests; Liofilchem, Roseto degli Abruzzi, Italy) with or without the presence of cloxacillin, was considered as positivity for AmpC overexpression [26,30]. Phenotypic screening for carbapenemase-production—performed if MER disk diameters were either in the intermediate (23–18 mm) or resistant (<19 mm) categories [28]—was assessed by the modified Hodge (cloverleaf) test (MHT), optimized for *P. aerugniosa*, as previously described [31]. In the assay, MER disks (10 µg; Oxoid, Basingstoke, UK) were utilized and *Escherichia coli* ATCC 25922 was used as an indicator organism.

### 2.5. Phenotypic Detection of Bacterial Efflux Pumps Contributing to the Drug-Resistant Phenotype

The effect of phenylalanine-arginine β-naphthylamide (PAβN)—a compound with well-known efflux pump inhibitory activity—on the susceptibility of tested antimicrobials was detected using the agar dilution method described previously [32]. The experiments were performed in case of isolates resistant to MER and/or CIP, based on the disk diffusion-based susceptibility tests. During the experiments, the concentration of PAβN was 40 µg/mL in the agar base; a two-fold decrease in MER and/or CIP MICs (determined by E-tests; Liofilchem, Roseto degli Abruzzi, Italy) in the presence of PAβN, compared to the MIC values without the inhibitor, was considered as positivity for efflux pump overexpression [32]. *P. aeruginosa* ATCC 27853 was used as a control strain.

### 2.6. Biofilm Production

The capacity of respective *P. aeruginosa* isolates to produce biofilm was assessed using a microtiter-plate based method previously described by Ramos-Vivas et al. [33]. In brief, overnight *P. aeruginosa* cultures (grown on Luria–Bertani [LB] agar) were inoculated into 5 mL of Luria-Bertani (LB) broth, and incubated overnight at 37 °C. The following day, 180 μL of LB broth and 20 μL of *P. aeruginosa* suspension (set at 10^6^ CFU/mL) were measured onto 96-well flat-bottomed microtiter plates, to a final volume of 200 µL, and incubated for 24 h at 37 °C. After the incubation period, the supernatants were discarded and the wells were washed three times using 200 µL of phosphate buffered saline (PBS; pH at 7.2), to remove planktonic cells that may interfere with the the interpretation of the results. After washing, the wells were fixed with 250 μL of methanol (Sigma-Aldrich, St. Louis, MO, USA) for 10 min and stained with a 1.0% crystal violet (CV; Sigma-Aldrich, St. Louis, MO, USA) solution for 15 min. Subsequently, the CV solution was discarded, and the wells were washed three times with purified water to remove excess stain. The contents of the wells were re-suspended in 250 μL of 33% *v/v*% glacial acetic acid (Sigma-Aldrich, St. Louis, MO, USA) and absorbance at 570 nm (OD_570_) was measured using a microtiter plate reader. All experiments were carried out in triplicate. The interpretation of the results was carried out based on the recommendations of Ansari et al., i.e., isolates were classified as weak/non-biofilm producers with OD_570_ values < 0.12, moderate biofilm producers with OD_570_ = 0.12–0.24, and strong biofilm producers with OD_570_ values > 0.24, respectively [34].

### 2.7. Motility (Swimming, Swarming, and Twitching) Assays

For motility assays, bacterial cultures grown overnight were diluted to a density of 10^5^ CFU/mL and transferred to Petri dishes containing Tryptic Soy Agar (TSA) medium, with different concentrations of agar (0.3%, 0.8% and 2% for swimming, swarming, and twitching motility, respectively). The bacterial suspension was transferred into the agar medium by puncture using a pipette tip (at 1/2 depth for swimming and swarming motility and at full depth for twitching motility) [35,36]. The plates were then incubated at 37 °C for 24 h (swimming and swarming motility) and 48 h (twitching motility). After the incubation period, the diameter of the growth zones (mm) were measured; in case of swimming and swarming motility, the measurements were made directly, while in case of twitching motility, the agar layer was removed and the bottom of the plates were stained directly with 0.01% CV solution (Sigma-Aldrich, St. Louis, MO, USA) [35,36]. All experiments were carried out in triplicate.

### 2.8. Production of Pyoverdine and Pyocyanin Pigments

An overnight grown bacterial culture was diluted to 10^5^ CFU/mL in TSB, transferred to 24-well culture plates (Sarstedt, Nümbrecht, Germany), and incubated at 37 °C for 48 h. Following incubation, each bacterial suspension was collected in an Eppendorf tube and centrifuged at 10,000 RPM. The supernatants were transferred to transparent 96-well plates for pyocyanin measurements, while they were transferred to black 96-well plates for pyoverdine measurements. The absorbance for pyocyanin (OD_686_) was measured at λ = 686 nm, while the fluorescence of pyoverdine at λ_ex/em_ = 395/460 nm (EM_460_ at EX_395_), using a microtiter plate reader [37,38]. All experiments were carried out in triplicate.

### 2.9. Statistical Analyses

Descriptive statistical analysis (including means with ranges and percentages to characterize data) was performed using Microsoft Excel 2013 (Microsoft Corp., Redmond, WA, USA). Independent sample t-tests were performed to compare measurements of OD_570_ (for biofilm production), growth zones (for swimming, swarming and twitching motility), OD_686_ (for pyocyanin production) and EM_460_ (for pyoverdine production) between MDR and non-MDR *P. aeruginosa* isolates. A correlation matrix was calculated to determine the association between the measurements (i.e., numerical data OD_570_, growth zones in mm, OD_686_ and EM_460_, respectively) corresponding to the expression of virulence-determinants. In addition, correlation between the presence of resistance against tested antibiotics and biofilm formation was also determined, where isolates received a score of 0/1 depending on the susceptibility/resistance to an antimicrobial, and 0/0.5/1, based on the classification of the biofilm-forming capacity (weak/moderate/strong) of the isolate, respectively. Based on the value of the Pearson-correlation coefficients (r), the relationship between the variables was determined as follows: of |r| < 0.3 were denoted as weak correlation, 0.3 < |r| < 0.5 as moderate correlation, and 0.5 < |r| < 0.85 as strong correlation [39]. Statistical analyses were performed with SPSS software version 22 (IBM Corp., Armonk, NY, USA).

## 3. Results

### 3.1. Antimicrobial Resistance of P. aeruginosa Isolates, Resistotyping

The antimicrobial resistance levels of the *P. aeruginosa* isolates included in the study were the following: CAZ 56.95% (n = 172), LEV 54.97% (n = 166), CIP 52.64% (n = 159), FEP 49.34% (n = 149), GEN 33.77% (n = 102), AMI 25.82% (n = 78), MER 23.17% (n = 70), IMI 21.19% (n = 64), and COL 1.66% (n = 5; MIC > 2 mg/L); overall, 44.04% (n = 133) of isolates were classified as MDR. The distribution of the various resistotypes detected among *P. aeruginosa* isolates are presented in Table 1.

### 3.2. Detection of AmpC Overexpression, Carbapenemase Production, and Efflux Pump Overexpression Using Phenotypic Methods

To ascertain the contribution of various resistance mechanisms in the drug resistance of the relevant *P. aeruginosa* isolates, phenotypic tests were utilized. Using the cloxacillin plate-based assay, AmpC overexpression—corresponding to a two-fold decrease in the CAZ MICs—was noted in 48.26% (n = 83; 27.48% overall) of isolates; CAZ resistance was characteristic to all (83/83) of these isolates, while 61/83, 13/83, and 11/83 were resistant to FEP, MER, and IMI, respectively. The MHT test was used to detect for the production of carbapenemases: the test was positive in 25.71% (n = 18; 5.96% overall) of cases, when non-susceptibility to MER (or both) was seen. The effects of efflux pump overexpression (based on the PAβN screening agar, demonstrated by the two-fold decrease in MICs in the presence of the inhibitor) were noted in 31.42% (n = 22; 7.28% overall) with regards to MER resistance and in 54.72% (n = 87; 28.8% overall) regarding CIP resistance, respectively. In the case of n = 3 isolates AmpC-hyperproduction and MHT-positivity, while in n = 6 isolates, efflux pump overexpression and MHT positivity were noted; in n = 3 isolates, efflux pump overexpression, AmpC hyperproduction, and MHT positivity were detected simultaneously.

### 3.3. Biofilm Formation and Expression of Virulence Factors (Pigments, Motility) among Non-MDR and MDR P. aeruginosa

Biofilm formation assays were carried out in a microtiter plate-based platform, using CV staining. Based on the OD_570_ measurements, 19.87% (n = 60) of the isolates were weak/non-biofilm producers, 20.86% (n = 63) were moderate biofilm producers, while 59.27% (n = 179) were strong biofilm producers, overall. The distribution of isolates with different biofilm-forming capacities did not show pronounced differences among the MDR and non-MDR groups (Table 2). Additionally, while numerical differences may be seen, MDR and non-MDR isolates did not show significant differences in expression of virulence factors (measured via our in vitro experiments), with the exception of pyocyanin production (OD_686_), which was shown to be higher among MDR isolates (0.371 ± 0.193 vs. non-MDR: 0.319 ± 0.191) (Table 2).

The correlation between the presence/absence (denoted as 0 and 1 in the analyses) of resistance to relevant antibiotics and biofilm-forming capacity (denoted as 0, 0.5 and 1 in the analyses for weak, moderate, and strong biofilm production, respectively) is presented in Table 3. Similarly to our previous findings (MDR/non-MDR), biofilm formation did not show significant correlation with the existence of resistance to any antibiotic tested, with |r| values consistently < 0.3, with the exception of CIP (r = 0.309; *p* > 0.05) and LEV (r = 0.324; *p* > 0.05). The correlation matrix on the phenotypic expression of biofilm formation and other virulence factors is presented in Table 4; no relevant correlations (with |r| values consistently < 0.1) were seen between the rate of biofilm formation and pigment production or motility.

## 4. Discussion, Review of the Literature

The increasing prevalence of MDR infections globally—especially in non-fermenting Gram-negative bacteria—may be considered as one of the hallmarks of the 21st century, especially when it comes to the procurement of safe and effective medical care [40,41]. The injudicious use of antibiotics and the lack of newly developed antibiotics are important hallmarks of this impending crisis [42,43]. Based on the projections of the “Burden of Antimicrobial Resistance Collaborative Group”, corresponding to the European Union (EU) and the European Economic Area (EEA), over 700,000 infections, >33,000 deaths, and ~900,000 disability-adjusted life years (DALY) were attributable to MDR bacteria, in the year 2015 alone [44]. Non-fermenting Gram-negative bacteria extensively contribute to the overall infectious disease burden—especially in immunocompromised individuals—which is now showing an increasing trend of morbidity and mortality (HAIs are associated with an overall mortality rate of 20–60%), due to the developments in resistance rates [45,46]. This has been confirmed by the World Health Organization’s (WHO) published report, in which carbapenem-resistant *Acinetobacter baumannii* complex (CR-AB), carbapenem-resistant *P. aeruginosa* (CR-PA), and carbapenem-resistant and/or ESBL-producing Enterobacterales were all identified as “critical priority” pathogens for the development of novel antibiotics and alternative anti-infective treatment strategies [47].

*P. aeruginosa*—especially under strong selection pressures in nosocomial environments—has the capacity of becoming resistant to most relevant antimicrobials [9,48]. This includes intrinsic non-susceptibility to many orally-available agents (e.g., a chromosomally-encoded AmpC β-lactamase, which may be stably de-repressed), and the acquisition of novel resistance genes through the means of horizontal gene transfer (i.e., integrons, plasmids, or transposons), leading to resistance to the four major group of anti-pseudomonal agents, namely relevant cephalosporins (and cephalosporin/β-lactamase combinations), carbapenems, aminoglycosides, respiratory fluoroquinolones (ciprofloxacin, levofloxacin, and moxifloxacin), and colistin [49]. Based on the data from the ECDC Surveillance Atlas of Infectious Diseases for 2019, resistance rates in *P. aeruginosa* ranged between 3.5−52.2%, 4.5–52.2%, 0.3–48.9% and 0–55.4% for ceftazidime, fluoroquinolones, aminoglycosides, and carbapenems in EU countries, respectively [50]. In addition to these genetically-determined resistance mechanisms (leading to the expression of mutated target proteins or inactivating enzymes, the relevance of so-called “adaptive” mechanisms also needs to be discussed; strong biofilm formation and metabolic downregulation towards the emergence of SCVs are difficult to study in vitro, but their relevance in in vivo infection dynamics must not be underestimated, as they lead to therapy-resistant, recalcitrant infections [51].

In clinical practice, MDR *Pseudomonas* infections are most often treated by carbapenem antibiotics, as these drugs are often considered the last safe and effective alternatives to treatment, especially in some patients (e.g., the elderly), where other drugs would be contraindicated due to their toxic adverse events or in cases (e.g., critically ill patients) where pathophysiological changes may significantly alter antibacterial pharmacokinetics, making the dosing of other drugs difficult [52]. Thus, the growing rate of CR-PA in nosocomial infections (with rates ranging between 0–60%, showing pronounced geographical differences) is a chilling concept; in a recently published meta-analysis, it was found that in addition to a history of carbapenem use, the prior use of piperacillin/tazobactam or vancomycin were all relevant risk factors for the acquisition of CR-PA [53,54]. Phenotypic carbapenem resistance is often due to a combination of more than one mechanism of resistance, which may be mediated via modifications in the transpeptidases (or penicillin-binding proteins; PPB2 and 3) [55], outer membrane impermeability (often due to OprD porin deficiency or loss) [56], overexpression of efflux pump systems [57], and the production of carbapenemases. Carbapenemases are β-lactamases with versatile hydrolytic capabilities; they can hydrolyze penicillins, cephalosporins, monobactams, and carbapenems [58]. Based on their biochemical characteristics and substrate profile, carbapenemases are classified into serine (including Ambler Class A [KPC], C [AmpC] and D [OXA-48-like family]) and metallo-β-lactamases (MBLs; Ambler Class B) [59]. These enzymes are often encoded by horizontally-transferable genes, which are often also associated with resistance determinants to other classes of antimicrobial drugs [60]. Currently, the propagation of carbapenemase-producing organisms, especially in non-fermenting Gram-negative bacteria, is a public health issue these resistance-determinants may rapidly disseminate, leading to outbreaks of bacteria with extensive resistance [61]. While Class A and D carbapenemases have also been described, the metallo-β-lactamases *bla*VIM and *bla*IMP are the most prevalent in *Pseudomonas* spp. [62,63]. The treatment of CR-PA infections heavily rely on last-resort agent that are either associated with more serious adverse events (colistin), or that are relatively new, with limited clinical experience in these infections (ceftolozane/tazobactam, ceftazidime/avibactam, meropenem/vaborbactam, plazmomycin) [64]. In addition, the therapy of metallo-β-lactamases is especially difficult, as currently there are no β-lactamase-inhibitors authorized for clinical use that would be effective against these enzymes. For example, in a study by O’Neall et al., n = 250 carbapenem non-susceptible *P. aeruginosa* isolates were tested for their susceptibility for ceftolozane/tazobactam and ceftazidime/avibactam, showing that these drugs presented with good in vitro efficacy against these isolates, but only as long as they did not produce carbapenemases; on the other hand, isolates carrying *bla*VIM or *bla*NDM were resistant to these new antibiotic combinations [65]. Thus, it is unsurprising that many clinical studies have highlighted that CR–PA infections are associated with worse clinical outcomes, compared to their carbapenem-susceptible counterparts [66].

In studies trying to ascertain the relationship between biofilm formation and drug resistance, phenotypic or a combination of phenotypic and molecular-based (PCR) methodologies are used, involving a wide variety of isolates from distinct clinical origins [67,68]. The majority of these studies have highlighted that around 75–99% of *Pseudomonas* spp. are biofilm producers, including 8–50% being characterized as strong biofilm producers [23]. As a part of our study, a large pool (n = 302) of *P. aeruginosa* isolates from diverse geographical and clinical origins were subjected to phenotypic tests to ascertain the possible correlation between biofilm-forming capacity, pigment production and motility (chosen as representative expressions of virulence), and antimicrobial resistance in these isolates. In our pool of isolates, resistance rates were the highest for ceftazidime and the fluoroquinolones, while >44% was classified as MDR. Over 25% isolates were characterized by the expression of AmpC β-lactamase, ~6% was positive for carbapenemase-production, and in ~7%, carbapenem non-susceptibility was affected by efflux pump overexpression, respectively (based on the phenotypic tests applied). Majority of isolates (59.27% overall) were strong biofilm producers, both in the MDR and non-MDR groups. Based on our results, no relevant correlation was seen between the resistance to individual antibiotics—or the presence of the MDR phenotype as a whole—and biofilm-forming capacity. Similarly—with the exception of pyocyanin production—no association was noted for other virulence factors and drug resistance. Based on our experimental data, we also did not observe a notable relationship between biofilm formation and other virulence factors. It is interesting to note that there was no association between the levels of different motilities expressed by *Pseudomonas*; this may be explained by the fact that—although all of these motility strategies have important physiological roles—their differential expression is needed to accommodate the adaptation of this pathogen to different niches [9,69].

Pyocyanin is a blue-green, lipid soluble pigment, which has redox-active properties, being a major electron acceptor for molecular O_2_; the synthesis of this pigment is mediated by PhzM (N-methyltransferase) and PhzS (flavin-dependent hydroxylase) [70]. This pigment has been described as an essential factor in the virulence of *Pseudomonas*, especially in skin and soft tissue infection and invasive infections of the lungs. Pyocyanin may often be detected in high concentrations in the airway tissues and sputum of CF patients, where it contributes to chronic lung infection and bronchiectasis by facilitating mucus overproduction, inhibition of ciliary activity and the α1-protease inhibitor (leading to excess tissue injury), and inhibition of IL-2 expression, leading to increased IL-8 release [71]. Pyocyanin may also have inhibitory properties on other bacterial species, aiding *Pseudomonas* in the competition in a given ecological niche; among others, this phenomenon was demonstrated in the decreased diversity of microbial communities in the sputa of patients, where pyocyanin concentrations were high [72,73]. Our experiments found that the production of pyocyanin was a distinctive hallmark between the MDR and non-MDR group, with isolates in the former group showing higher levels of pigment production. During the experiments of Fuse et al., reverse-phase high-performance liquid chromatography (RP-HPLC) was used for the quantitative extraction of pyocyanine in *P. aeruginosa*; their study concluded that MDR (including many MBL-producers) reduced pyocyanine expression, compared to the non-MDR group [74]. With similar aims to our study, Gholami et al. included 100 *P. aeruginosa* isolates from clinical and environmental origins; they found *bla*TEM and *bla*SHV positivity in 92%/16% and 20/6% of the isolates, respectively, with clinical isolates having higher proclivity to becoming MDR. 70% and 28% (with 98% and 70% of these isolates carrying the *algD* and *algU* genes) of these isolates were classified as strong biofilm producers; however, no association between the MDR phenotype and biofilm-formation was found [75]. Yamani et al. utilized phenotypic and genotypic methods to characterize 66 *Pseudomonas* isolates: in their study, 53.03%/24.24%/22.73% of isolates were classified as strong/moderate/weak biofilm producers, respectively. By measuring the expression levels of 10 virulence genes (*exoT*, *exoS*, *exoU*, *phZ*, *las* and *pil* genes), they found that—although all associated genes were present in all isolates—all biofilm and virulence-associated genes were significantly upregulated in non-MDR isolates, demonstrating an inverse correlation [76]. The experiments of Kamali et al. included 80 isolates, from which 50% were characterized as moderate or strong biofilm producers; they found that 87.5% possessed all three genes relevant for biofilm formation (*algD*, *pslD*, and *pelF*) and a positive correlation between biofilm formation and the presence of these genes, while no association was noted between biofilm formation and drug resistance [77]. Jabalameli et al. studied the carriage of Type III secretory toxin-encoding genes and their cytotoxic action on A549 human lung cancer cell lines in 96 clinical *P. aeruginosa* isolates, finding that the carriage of *exoT*, *exoY*, *exoU,* and *exoS* were 100%, 95%, 64.5%, and 29%, respectively. 47%/26% of the isolates were strong/moderate biofilm producers, respectively; no association was seen between resistance phenotype and biofilm formation, and interestingly, neither between the presence of toxin-encoding genes and the extent of toxic effects seen on the cell lines [78].

In contrast, the study of Abidi et al., which included 22 *P. aeruginosa* isolated from keratitis, concluded that strong biofilm producers were significantly more common among MDR isolates [79]. Choy et al. involved 77 *Pseudomonas* isolates originating from keratitis of contact lens and non-contact lens origin; while no correlation was seen between drug resistance and biofilm formation, the presence of the *exoU* gene (mostly found in contact lens-related isolates) and strong biofilm formation showed strong positive correlations [80]. In a study involving 74 *P. aeruginosa* from the sputum of CF and non-CF patients, Perez et al. noted that no differences in biofilm formation were observed among isolates from different patient origins (although 85.7% of CF isolates presented as the mucoid phenotypic variant), MBL positivity showed strong association with strong biofilm production [81]. In a study involving n = 190 isolates, Eladawy et al. found no relevant associations between antimicrobial resistance, biofilm formation, and the presence of genes encoding for selected virulence factors (i.e., alignate, biofilm, exotoxin A, L-ornithine monooxygenase, phospholipases T3SS, proteases, and pyocyanin) [82]. da Costa Lima et al. used molecular methods to assess the correlation between the presence of quorum sensing (QS) genes and biofilm formation in 40 *P. aeruginosa* isolates: while all isolates carried these genes, no association was found with the capacity to produce biofilm [83]. Subedi et al. included 22 isolates from eye infections and CF sputa for testing: the prevalence of *exoU* was 61.5%, and isolates carrying exoU showed higher rates of resistance and higher rates of mutations in the quinolone resistance determining region (QRDR) [84]. In a study involving 78 clinical and environmental isolates, Karami et al. found MDR rates of 48.7% and a strong positive association was shown between biofilm-formation and the MDR status [85]. Bahador et al. noted a positive association between biofilm formation and the presence of the *exoU* and *exoS* genes (36.6% and 55.7%, respectively), but not with antimicrobial resistance (16% of isolates were extensively drug resistant [XDR]) in the 75 pseudomonads tested [86]. In a comprehensive laboratory study by Milojkovic et al. involving 94 isolates—similarly to our study—no correlation was noted between antibiotic resistance, genetic composition, pigment production, serotypes, and biofilm formation [87]. Cho et al. assessed biofilm formation and MBL carriage in carbapenem-resistant *P. aeruginosa* isolates; the overwhelming majority of isolates (>92%) were biofilm producers. Furthermore, the presence of the *pslA* gene and the MDR phenotype all showed positive association with biofilm-forming capacity [88]. Eighty-six *P. aeruginosa* isolates (involving >31% MDR and >12% XDR), collected from various clinical materials, were included in the study of Zahedani et al., where strong correlations were found between the expression of efflux pumps and biofilm formation (22.47%/21.34%/11.23% were strong/moderate/weak biofilm producers); additionally, the presence of some efflux pumps (MexEF-OprN) reduced the virulence of these isolates, while others (MexAB-OprM, MexCD-OprJ) did not have the same effect [89]. Rodulfo et al. involved n = 176 strains (isolated between 2009 and 2016) in their experiments, showing high levels of drug resistance (38.1% MDR), and resistance rates showed strong association with the presence of *exoU* and hemolysin, while negative associations with twitching motility [90]. In isolates originating from endotracheal aspirates from ICU patients, Fricks-Lima et al. found strong and significant correlations between ceftazidime and imipenem-resistance and strong biofilm-forming capacity [91].

Biofilms are characterized by a protective form of growth (essential for survival) in a nosocomial environment, which may also maintain an inflammatory environment in vivo [91,92]. Nevertheless, it is obvious that the continuous, long-term expression of antibiotic resistance-determinants and virulence factors is detrimental for the microorganism’s ability to adapt to environmental changes [93,94]. For example, in chronic infections, where *P. aeruginosa* establishes long-term persistence in biofilm, the expression of virulence factors is downregulated, to accommodate for the lower metabolic activity in the exopolysaccharide matrix [95]. QS is one of the principal mechanisms responsible for—among others—the regulation of virulence factor expression and biofilm formation. QS in *Pseudomonas* consist of a three interconnected systems, namely the Las, Rhl, and Pqs systems; these systems facilitate the changes needed for the pathogens’ survival, by the sensing of diffusible signal molecules found in their surroundings, which is a proxy for the population density in the niche [96,97,98]. However, many antibiotics also have the ability to affect these QS systems in *Pseudomonas*, either by directly acting on gene expression or by the degradation of these signal molecules [99]. The emergence of *P. aeruginosa* isolates with different biofilm-forming capacities may also inform the genetic heterogeneity within these species, which is an important factor for successful infection in humans [9]. Nevertheless, differences in the phenotypes and susceptibility trends in these isolates may also stem from their different geographical origin and the sampling frame used [100]. The main limitation of our study was the reliance on the use of phenotypic methods only during the assessment of the resistance, biofilm-forming, and virulence phenotype of the isolates; therefore, we did not have data from molecular methods or on the clonal complexes (CCs) for the isolates implicated in the assays. On the other hand, our data are generated from a considerably large number of geographically-distinct isolates.

## 5. Conclusions

*P. aeruginosa* is an opportunistic pathogen, which is often implicated in infectious pathologies, leading to a considerable disease burden worldwide. Along with its intrinsic resistance to many antibiotics, *Pseudomonas* infections are further complicated by the increasing prevalence of carbepenem and colistin non-susceptibility in these strains. *Pseudomonas* species possess many important virulence factors in their repertoire, in addition to their propensity to form biofilms (75–100% are biofilm producers). Data on interplay between the presence and mechanisms of drug resistance with those of biofilm formation and virulence is crucial to address chronic bacterial infections (leading to therapeutic failure and decreased quality of life in the affected patients), and to provide strategies for their management. Our study has showed that isolates with the MDR and non-MDR phenotype did not differ significantly in the context of the virulence determinants studied (biofilm, various motilities, pyoverdine), with the exception of pyocyanin production. Similarly, no statistically-relevant interplay was observed between the individual virulence factors. The latter may either be explained by the adaptation of the microorganism to express their virulence factors only in situations where they are necessary for survival, or our in vitro methodologies utilized were not sophisticated enough to detect their association. As presented in our paper, many authors have aimed to provide clarity on association between biofilm-forming capacity, virulence factor-expression, and the MDR phenotype in *P. aeruginosa*, but the presently-available results do not yet allow for a conclusion to be made. Additional experiments—preferably involving genomics and/or in vivo methods—are needed to provide further clarity and insights into this field.

## Figures and Tables

**Table 1:** Resistotype distribution and MAR indices of respective isolates.

Resistotypes	Resistance Pattern	MAR Index	MDR Status	Ratio of Isolates (n, %)
0	None	0	non-MDR 55.96% (n = 169)	130 (43.05%)
I	CAZ	0.111		1 (0.33%)
II	CAZ, LEV	0.222		7 (2.32%)
III	CAZ, CIP, LEV	0.333		15 (4.97%)
IV	CAZ, FEP, CIP	0.333		4 (1.32%)
V	CAZ, FEP, LEV	0.333		4 (1.32%)
VI	CAZ, FEP, GEN	0.333		5 (1.66%)
VII	CAZ, FEP, CIP, LEV	0.444		3 (0.99%)
VIII	CAZ, FEP, CIP, LEV, GEN	0.555	MDR 44.04% (n = 133)	5 (1.66%)
IX	CAZ, FEP, CIP, LEV, IMI	0.555		10 (3.31%)
X	CAZ, FEP, CIP, LEV, MER	0.555		10 (3.31%)
XI	CAZ, FEP, CIP, LEV, GEN, IMI	0.666		7 (2.32%)
XII	CAZ, FEP, CIP, LEV, GEN, MER	0.666		7 (2.32%)
XIII	CAZ, FEP, CIP, LEV, IMI, MER	0.666		16 (5.29%)
XIV	CAZ, FEP, CIP, LEV, GEN, AMI	0.666		23 (7.62%)
XV	CAZ, FEP, CIP, LEV, GEN, AMI, IMI	0.777		16 (5.29%)
XVI	CAZ, FEP, CIP, LEV, GEN, AMI, MER	0.777		22 (7.28%)
XVII	CAZ, FEP, CIP, LEV, GEN, AMI, COL	0.777		2 (0.67%)
XVIII	CAZ, FEP, CIP, LEV, GEN, AMI, IMI, MER	0.888		12 (3.97%)
XIX	CAZ, FEP, CIP, LEV, GEN, AMI, IMI, MER, COL	1.000		3 (0.99%)

**Table 2:** Biofilm formation and expression of other virulence factors among non-MDR and MDR *P. aeruginosa.*

	Non-MDR (n = 169)	MDR (n = 133)	Statistics
Biofilm formation (OD_560_) (mean ± SD)	0.333 ± 0.216	0.316 ± 0.200	n.s. (*p* = 0.634)
Weak/non-biofilm producer (n, %)	18.34% (n = 31)	21.80% (n = 29)	n.r.
Moderate biofilm producer (n, %)	30.77% (n = 52)	8.27% (n = 11)	
Strong biofilm producer (n,%)	50.89% (n = 86)	69.93% (n = 93)	
Swimming motility (mm) (mean ± SD)	29.31 ± 10.99	27.46 ± 10.38	n.s. (*p* = 0.183)
Swarming motility (mm) (mean ± SD)	32.05 ± 17.88	32.79 ± 18.49	n.s. (*p* = 0.728)
Twitching motility (mm) (mean ± SD)	13.63 ± 7.24	14.23 ± 6.75	n.s. (*p* = 0.458)
Pyocyanin production (OD_686_) (mean ± SD)	0.319 ± 0.191	0.371 ± 0.193	***p = 0.018***
Pyoverdine production (EM_460_) (mean ± SD)	2012 ± 1132	2064 ± 1124	n.s. (*p* = 0.774)

n.s.: not significant; n.r.: not relevant; values in boldface were *p* < 0.05.

**Table 3:** Correlation matrix regarding the microbial categories (weak/moderate/strong) based on biofilm formation and resistance to antibiotics in *P. aeruginosa* isolates.

	CAZ	FEP	IMI	MER	CIP	LEV	GEN	AMI	COL	
**Biofilm formation**	0.123	0.134	0.101	0.124	0.309	0.324	0.139	0.113	−0.012	Pearson-correlation coefficient (r = )
	0.231	0.218	0.581	0.564	0.102	0.099	0.567	0.605	0.986	Statistics (*p* = )

**Table 4:** Correlation matrix regarding the phenotypic expression of biofilm formation and other virulence factors among tested *P. aeruginosa* isolates.

	Biofilm Formation (OD_570_)	Swimming Motility (mm)	Swarming Motility (mm)	Twitching Motility (mm)	Pyocyanin Production (OD_686_)	Pyoverdine Production (EM_460_)	
**Biofilm formation (OD_570_)**	X	0.981	0.580	0.518	0.581	0.373	Statistics (*p* = )
**Swimming motility (mm)**	−0.003	X	0.143	0.432	0.998	0.244	
**Swarming motility (mm)**	0.032	0.084	X	0.303	0.351	0.389	
**Twitching motility (mm)**	−0.032	−0.045	−0.059	X	0.118	0.244	
**Pyocyanin production (OD_686_)**	−0.0370	0.001	0.054	0.090	X	0.814	
**Pyoverdine production (EM_460_)**	0.051	−0.067	−0.050	−0.067	0.014	X	
	Pearson correlation coefficient (r = )						

## Data Availability

All data generated during the study are presented in this paper.
